# Structural insight into activity enhancement and inhibition of H64A carbonic anhydrase II by imidazoles

**DOI:** 10.1107/S2052252514004096

**Published:** 2014-02-28

**Authors:** Mayank Aggarwal, Bhargav Kondeti, Chingkuang Tu, C. Mark Maupin, David N. Silverman, Robert McKenna

**Affiliations:** a Department of Biochemistry and Molecular Biology, University of Florida, PO Box 100245, Gainesville, FL 32610, USA; b Department of Pharmacology and Therapeutics, University of Florida, PO Box 100247, Gainesville, FL 32610, USA; c Department of Chemical and Biological Engineering, Colorado School of Mines, 1500 Illinois Street, Golden, CO 80401, USA

**Keywords:** human carbonic anhydrase, H64A, activity enhancement, rescue, activation, imidazole

## Abstract

Human carbonic anhydrases (CAs) are zinc metalloenzymes that catalyze the hydration and dehydration of CO_2_ and HCO_3_^−^, respectively. The reaction follows a ping-pong mechanism, in which the rate-limiting step is the transfer of a proton from the zinc-bound solvent (OH^−^/H_2_O) in/out of the active site *via* His64, which is widely believed to be the proton-shuttling residue. The decreased catalytic activity (∼20-fold lower with respect to the wild type) of a variant of CA II in which His64 is replaced with Ala (H64A CA II) can be enhanced by exogenous proton donors/acceptors, usually derivatives of imidazoles and pyridines, to almost the wild-type level. X-ray crystal structures of H64A CA II in complex with four imidazole derivatives (imidazole, 1-­methylimidazole, 2-­methylimidazole and 4-methylimidazole) have been determined and reveal multiple binding sites. Two of these imidazole binding sites have been identified that mimic the positions of the ‘in’ and ‘out’ rotamers of His64 in wild-type CA II, while another directly inhibits catalysis by displacing the zinc-bound solvent. The data presented here not only corroborate the importance of the imidazole side chain of His64 in proton transfer during CA catalysis, but also provide a complete structural understanding of the mechanism by which imidazoles enhance (and inhibit when used at higher concentrations) the activity of H64A CA II.

## Abstract 2

Human carbonic anhydrases are zinc metalloenzymes that catalyze the hydration and dehydration of CO_2_ and HCO_3_^−^, respectively. X-ray crystal structures of a variant of human carbonic anhydrase II in complex with four imidazole derivatives (imidazole, 1-methylimidazole, 2-methylimidazole and 4-methylimidazole) have been determined in order to identify the binding sites for such compounds, and a mechanism to explain the effects on catalytic activity is proposed.

## 1. Introduction   

Human carbonic anhydrase (CA) II is a ∼29 kDa monomeric soluble cytosolic isoform that is found predominantly in red blood corpuscles and has a *k*_cat_/*K*_m_ of 1.5 × 10^8^ *M* ^−1^ s^−1^ (Supuran *et al.*, 2003 ▶; Supuran, 2008 ▶). The catalytic CAs are involved in various physiological reactions, including respiration, pH regulation, Na^+^ retention, calcification, tumori­genesis, electrolyte secretion, gluconeogenesis, ureagenesis and lipogenesis (Vullo *et al.*, 2003 ▶, 2004 ▶; Vullo, Innocenti *et al.*, 2005 ▶; Vullo, Voipio *et al.*, 2005 ▶; Lehtonen *et al.*, 2004 ▶; Supuran *et al.*, 2004 ▶; Nishimori *et al.*, 2005*a* ▶,*b* ▶; Nishimori, Minakuchi, Onishi, Vullo, Cecchi *et al.*, 2007 ▶; Nishimori, Minakuchi, Onishi, Vullo, Scozzafava *et al.*, 2007 ▶; Alterio *et al.*, 2006 ▶).

**Figure 1:**
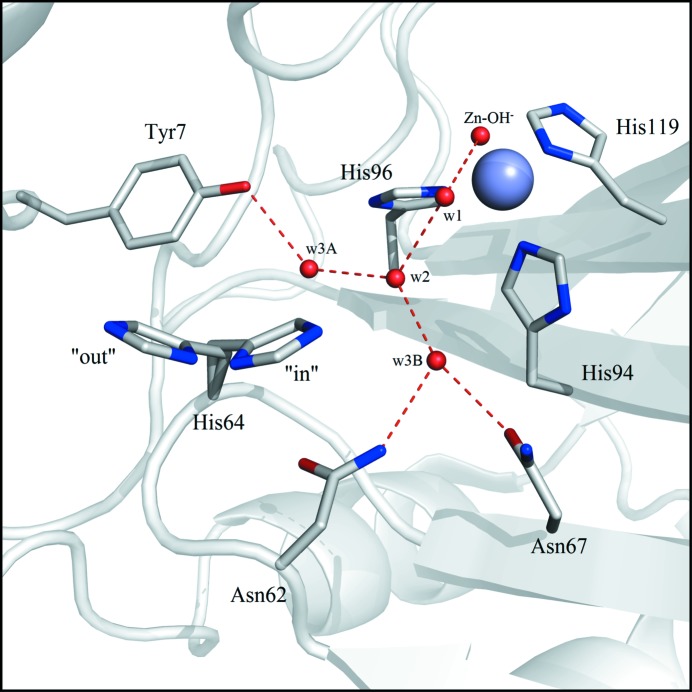
Active site of CA II at pH 8.0 (PDB entry 3ks3; Avvaru *et al.*, 2010 ▶). The zinc ion and water molecules are shown as light blue and red spheres, respectively. Hydrogen bonds are represented as dotted red lines. Residues are as labeled.

The catalysis relies on a zinc hydroxide/water (ZnOH^−^/H_2_O) mechanism to reversibly convert CO_2_ to HCO_3_^−^ and a proton. The first step of catalysis, in the hydration direction, is a nucleophilic attack on an incoming CO_2_ by the zinc-bound OH^−^ (ZnOH^−^) to produce HCO_3_^−^. The binding of HCO_3_^−^ at the metal is weak and is accordingly displaced by a water molecule (1). The second step of the reaction, which is rate-limiting, is the transfer of a proton from the zinc-bound water to regenerate the ZnOH^−^. This is thought to occur through a network of well ordered hydrogen-bonded waters in the active site, and to be facilitated by the protonation/deprotonation of the imidazole side chain of His64 (2) (Fig. 1 ▶).
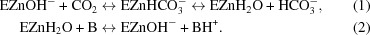



X-ray crystallographic studies (Nair & Christianson, 1991 ▶) and molecular-dynamics (MD) calculations (Maupin & Voth, 2007 ▶; Maupin *et al.*, 2011 ▶) have shown the side chain of His64 to exhibit two conformations in CA II at pH 7.8. The rotation about the C^α^—C^β^ bond of His64 has a Δ*G* of ∼6.0 kcal mol^−1^ (equivalent to a rate of 2.45 × 10^8^ s^−1^; Maupin *et al.*, 2009 ▶, 2011 ▶); hence, its conformational switching can occur on a time scale as fast as the catalysis. A possible mechanism for proton transfer is that His64 accepts a proton in an inward orientation pointing towards the active site, and delivers it to the bulk solution by flipping into the outward orientation pointing away from the active site during the second step of the hydration reaction (2). It has been postulated that the inward conformation of His64 is poised to accept the excess proton from the water network, while the outward conformation is ready for proton shuttling to the bulk solvent. This observed dual conformer of His64 is most likely to be related to its protonation state (Fisher *et al.*, 2011 ▶). Exactly how protons move in the active site of proteins is not clear, but it has been proposed that a Grotthus proton-hopping mechanism or the formation of a series of Zundel (H_5_O_2_^+^) or Eigen (H_9_O_4_^+^) cations may be involved (Elder *et al.*, 2005 ▶; Maupin *et al.*, 2009 ▶, 2011 ▶). The enzyme displays very a strong pH dependence of both *k*_cat_ and *k*_cat_/*K*_m_ that are defined by a p*K*_a_ of ∼7 (Tu *et al.*, 1989 ▶; Lindskog, 1997 ▶; Fisher *et al.*, 2007 ▶; Roy & Taraphder, 2007 ▶; Silverman & McKenna, 2007 ▶).

When His64 is mutated to Ala (H64A) in CA II, it causes a reduction in the enzymatic proton shuttling and thus decreases the rate of reaction by ∼20-fold (Tu *et al.*, 1989 ▶). This decrease in catalysis has been shown to be rescued in a saturable manner by the addition of exogenous proton acceptors in solution, such as histamine, small imidazoles, pyridines and their derivatives (Tu *et al.*, 1989 ▶; An *et al.*, 2002 ▶; Dave *et al.*, 2011 ▶; Saada, Montero *et al.*, 2011 ▶; Saada, Vullo *et al.*, 2011 ▶). It has been an intriguing observation that the activity enhancement of H64A CA II by these external proton donors/acceptors is substantial, with the rate of catalysis at saturation levels of certain imidazole and pyridine derivatives approaching that of wild-type CA II (Duda *et al.*, 2001 ▶).

Only two binding sites for such enhancers (Briganti *et al.*, 1997 ▶; Duda *et al.*, 2001 ▶; An *et al.*, 2002 ▶) have been identified by X-ray crystallography (Duda *et al.*, 2001 ▶) and NMR (Elder *et al.*, 2005 ▶) within and around the active-site cavity of H64A CA II, and it is unclear whether or not these sites are indicative of the proton-transfer pathway. For example, X-ray diffraction of H64A CA II crystals soaked in 4-methylimidazole (4MI) showed a binding site 12 Å away from the zinc ion, making a π-stack with Trp5 in the active-site cavity (Duda *et al.*, 2001 ▶); however, this binding site was not productive in proton transfer in catalysis as demonstrated by the subsequent kinetic analysis of a W5A CA II variant (An *et al.*, 2002 ▶) and multi-state empirical valence-bond simulations (Maupin *et al.*, 2011 ▶).

## 2. Experimental procedures   

### 2.1. Expression and purification of H64A CA II   

The recombinant gene for H64A CA II was cloned into a pET-32b plasmid vector with an ampicillin-resistance gene and expressed in *Escherichia coli* BL21 (DE3) cells. The culture was grown at 37°C in the presence of ampicillin (100 µg ml^−1^) until it reached an OD_600_ of 0.6, and was thereafter induced with IPTG (100 µg ml^−1^) for protein expression. The lysate was then purified by affinity chromatography using a *p*-­aminomethylbenzenesulfonamide column. Nonspecifically bound proteins were washed off the column using buffers (200 m*M* sodium sulfate) at pH 7.0 and pH 9.0, finally eluting the protein with elution buffer (400 m*M* sodium azide). The enzyme was thereafter buffer-exchanged to remove sodium azide and concentrated to 15 mg ml^−1^ (450 µ*M*) using a 10 kDa filter. The detailed vector preparation and protein purification has been published previously (Khalifah *et al.*, 1977 ▶; An *et al.*, 2002 ▶).

### 2.2. Co-crystallization and X-ray data collection of CA complexes   

Co-crystals of each of the H64A CA II–compound complexes [with the small imidazoles imidazole (I), 1-methylimidazole (1MI), 2-methylimidazole (2MI) and 4-methylimidazole (4MI)] were obtained using the hanging-drop vapor-diffusion method. Drops of 10 µl (0.3 m*M* protein, 100 m*M* small imidazole, 0.8 *M* sodium citrate, 50 m*M* Tris–HCl pH 8.0) were equilibrated against the precipitant solution (1.6 *M* sodium citrate, 50 m*M* Tris–HCl pH 8.0) at room temperature (298 K) for all of the small imidazoles. Crystals were observed after 5 d and, based on visual selection, a crystal of each of the CA complexes was cryoprotected by quick immersion into 20% sucrose precipitant solution and flash-cooled by exposure to a gaseous stream of nitrogen at 100 K. The X-ray diffraction data for the I, 1MI and 4MI complexes were collected on the F1 beamline at the national X-ray facility at Cornell High Energy Synchrotron Source (CHESS). The crystal-to-detector distance was maintained at 100 mm, with 1° oscillation steps and 1 s exposure per image. Diffraction data for the 2MI complex were collected in-house with an R-­AXIS IV^++^ image-plate system on a Rigaku RU-H3R Cu rotating-anode generator operating at 50 kV and 22 mA using Osmic VariMax HR optics. Setting the crystal-to-detector distance to 80 mm and using oscillation steps of 1°, a 5 min exposure time was used per image.

### 2.3. Structure determination of CA–drug complexes   

X-ray diffraction data indexing, integration and scaling were performed using *HKL*-2000 (Otwinowski & Minor, 1997 ▶). Starting phases were calculated from PDB entry 3ks3 (Avvaru *et al.*, 2010 ▶) with waters removed. Refinement using the *PHENIX* package (Adams *et al.*, 2010 ▶), with 5% of the unique reflections selected randomly and excluded from the refinement data set for the purpose of *R*_free_ calculations (Brünger, 1992 ▶), were alternated with manual refitting of the model in *Coot* (Emsley & Cowtan, 2004 ▶). The validity of the final model was assessed by *PROCHECK* (Vaguine *et al.*, 1999 ▶)*.*

### 2.4. Ligand-binding studies using *PDBePISA*   

The buried surface areas of all four of the imidazoles in their respective crystal structures were calculated using the online *Proteins, Interfaces, Surfaces and Assemblies* algorithm provided by the Protein Data Bank in Europe (*PDBePISA*; Krissinel & Henrick, 2007 ▶). The Δ*G* values provided by this algorithm indicate the solvation free-energy gain upon formation of the interface in kcal mol^−1^. However, this value does not include the effect of satisfied hydrogen bonds and salt bridges across the interface.

## 3. Results and discussion   

**Figure 2:**
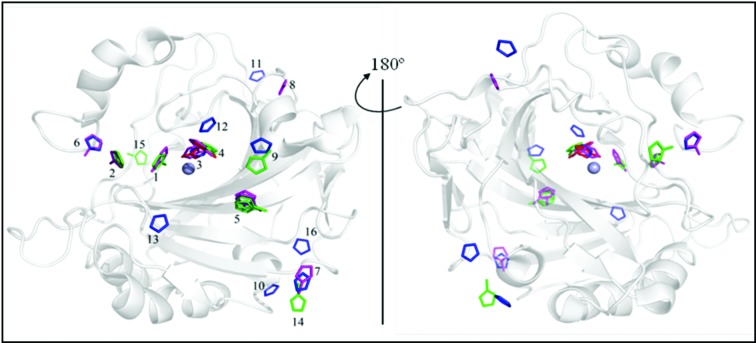
Overview of the 15 identified binding sites for imidazole (I; red sticks), 1-methylimidazole (1MI; green sticks), 2-methylimidazole (2MI; red sticks) and 4-methylimidazole (4MI; magenta sticks) in and around H64A CA II. The numbers represent site identifications (as given in Table 2 ▶).

**Figure 3:**
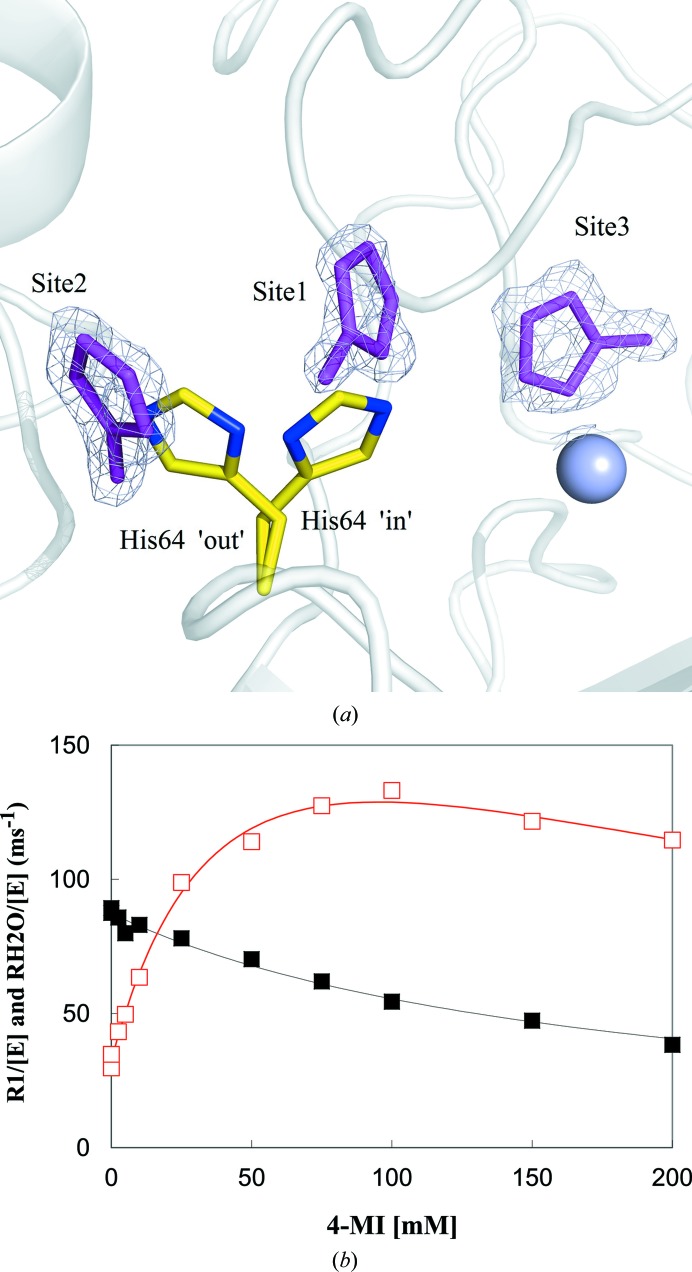
(*a*) Binding sites (sites 1–3) of 4-methylimidazole (4MI) within and near the active site of H64A CA II. 2*F*_o_ − *F*_c_ electron-density maps are contoured at 1.2σ. Zn is shown as a blue sphere and His64 from wild-type CA II (PDB entry 3ks3; Avvaru *et al.*, 2010 ▶) is shown as yellow sticks to represent the ‘in’ and ‘out’ conformations. (*b*) The inhibition by 4MI of CO_2_/HCO_3_^−^ interconversion (*R*_1_/[E], black) and enhancement of proton transfer (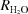/[E], red) on H64A CA II.

Each of the four imidazole compounds I, 1MI, 2MI and 4MI were co-crystallized with H64A CA II, the structures were determined to a resolution of at least 1.7 Å (Table 1 ▶) and the coordinates were deposited in the Protein Data Bank as PDB entries 4hf3 (I), 4hez (1MI), 4hew (2MI) and 4hey (4MI). All four structures had a main-chain r.m.s.d. of <0.2 Å when compared with wild-type CA II (PDB entry 3ks3; Avvaru *et al.*, 2010 ▶), demonstrating no significant structural perturbation of the enzyme. OMIT *F*_o_ − *F*_c_ electron-density maps were calculated and the respective imidazoles were located, fitted and refined. Based on where these compounds were located in the structure, 15 unique sites were characterized (Fig. 2 ▶ and Table 2 ▶). Of these 15 sites, three were identified as of significant importance in affecting catalysis (the sites labeled 1–3 and shown for 4MI in Fig. 3 ▶ *a*) based on their location within the active site. Sites 1 and 2 were defined as the overlapping regions of the inward and outward conformations, respectively, of His64 in wild-type CA II. The binding of imidazoles in sites 1 and 2 provides a possible proton-donor/acceptor group within these locations, thereby restoring the reduced proton-shuttle capability of the enzyme. Site 3 overlaps with the CO_2_-binding site (Domsic *et al.*, 2008 ▶), displacing the ZnOH^−^. Thus, the binding of any imidazole molecule in this region would suggest enzyme inhibition, as the ZnOH^−^ is essential for catalysis. The other sites mostly appear on the surface of the enzyme (sites 5, 7, 9–11 and 13–16); being this far from the zinc, it is improbable that they affect catalysis.

### 3.1. Imidazole (I)   

A total of nine molecules of I were bound to H64A CA II, with an average *B* factor of 24.5 Å^2^, which is comparable to the *B* factors of surrounding solvent molecules and residues (Table 2 ▶ *a*). The absence of a methyl group (compared with the other imidazoles in this study) imparts low hydrophobicity to I (log *P* = −1.0, where *P* is the water/oil partition coefficient), thereby reducing the possibility of it being involved in hydrophobic interactions. This could explain why I was not observed at sites 1 and 2. At concentrations as high as 100 m*M* in the crystallization conditions, I was also bound to the active-site Zn^2+^ (site 3 in Fig. 3 ▶), forming a very stable coordination geometry (Δ*G* = −12.4 kcal mol^−1^) displacing four water molecules including the ZnOH^−^, indicating inhibition. Kinetics data showing this inhibition are provided in the Supporting Information (Supplementary Fig. S1).

### 3.2. 1-Methylimidazole (1MI)   

1MI was bound at seven different sites (average *B* factor of 26.0 Å^2^). Although 1MI and I have similar p*K*_a_ values (p*K*_a_ ≃ 7.1), the presence of a methyl group makes 1MI more hydrophobic in nature and possibly leads to its ‘capture’ at sites 1 and 2 (Table 2 ▶ *b*). There were various other sites (inside the protein and out on the surface) where 1MI molecules were located, but it is improbable that they affect the catalysis of H64A CA II. 1MI binds to the active-site zinc with its unmethylated N atom, and the other methylated N atom lacks the ability to form hydrogen bonds to Thr200, which could explain its absence at site 3.

### 3.3. 2-Methylimidazole (2MI)   

There were only two molecules of 2MI (p*K*_a_ = 8.2) bound to H64A CA II (Supplementary Fig. S2*a*), both with an average *B* factor of 19.2 Å^2^ and in close proximity to each other, interacting with the active-site residues Val121, Phe131, Leu198, Thr199 and Pro202. The computed Δ*G* values were higher, most likely because of a coordination geometry between imidazole and Zn^2+^ at site 3 which makes this site the most stable. Occupying both sites 3 and 4, 2MI buried a total surface area of 190.3 Å^2^ (Supplementary Fig. S2*b* and Table 2 ▶ *c*). Inhibition data on 2MI are shown in Supplementary Fig. S2(*a*).

### 3.4. 4-Methylimidazole (4MI)   

With an average *B* factor of 20.8 Å^2^, 4MI showed perhaps the most complete binding profile among all the imidazoles examined, providing structural insights into how an imidazole ring can enhance or inhibit the activity of H64A CA II. 4MI is the most hydrophobic of all four of the compounds (log *P* = 0.3) and has two N atoms that can participate in hydrogen bonds which help 4MI bind at a maximum number of sites. It occupied sites 1 and 2, which readily offers a structural explanation of how 4MI is able to enhance the activity of H64A CA II by acting as the external proton donor/acceptor for either or both of the ‘in’ and ‘out’ conformations of His64 (Table 2 ▶ *d*). Another molecule of 4MI, similar to I and 2MI, was observed to be directly bound to the active-site Zn^2+^, displacing four water molecules including ZnOH^−^ (Fig. 3 ▶ *a*). This displacement of ZnOH^−^ by 4MI clearly suggests the mechanism of inhibition of CA activity by high concentrations of the compound (Fig. 3 ▶ *b*).

## 4. Conclusion   

We have co-crystallized four small imidazoles, imidazole (I), 1-­methylimidazole (1MI), 2-methylimidazole (2MI) and 2-­methylimidazole (4MI), with H64A CA II to identify the binding sites for such compounds and propose a mechanism to explain the effects on catalytic activity. This is the first X-ray crystal structure that reports simultaneously (i) an inhibitory binding site for imidazoles (directly bound to the active-site zinc) and (ii) sites occupied by both the ‘in’ (not previously observed) and ‘out’ conformations of His64 in wild-type CA II. Previously published enzyme-kinetics studies (Duda *et al.*, 2001 ▶; An *et al.*, 2002 ▶) to analyze the CA activity enhancement effects of these imidazoles were performed again using an ^18^O-exchange method and are included in the Supporting Information. Additionally, the effects of imidazole on wild-type CA II and H64A CA II have also been studied previously, in which the inhibition profiles appeared to be identical for both of them (Taoka *et al.*, 1994 ▶). Imidazole shows a large activity enhancement of H64A CA II but a very weak enhancement of wild-type CA II (Taoka *et al.*, 1994 ▶).

From our kinetic studies, it is clear that as the concentrations of imidazoles and their derivatives increase their effect on the catalytic activity of H64A CA II changes from enhancement to inhibition (Fig. 3 ▶ *b*; 4MI example; Supplementary Figs. S1–S3). The mechanism behind this enhancement of activity and inhibition has long been discussed. Site 2 is the only site that has previously been found to be occupied by 4MI (Duda *et al.*, 2001 ▶). However, mutation studies on Trp5 (which was initially thought to stabilize the binding of 4MI by π-stacking) showed that activity enhancement of H64A CA II by 4MI occurred regardless of the presence of Trp5. Moreover, since the bound 4MI corresponded to only one (outward) conformation of His64 (the inward conformation is equally important for proton shuttling to be effective), it was not considered enough of a structural proof to be able to make a conclusion on the role of 4MI in activity enhancement (An *et al.*, 2002 ▶).

We present the first structural evidence which provides an explanation of this phenomenon. As the data show, there are not one but many regions where these compounds bind. Most of the sites are predominantly composed of hydrophobic amino acids (sites 3–4, 7–10 and 12–14); however, the presence of a methyl group on imidazole (1MI, 2MI and 4MI) does not seem to affect the hydrophobic interactions enough to cause changes in binding orientations or affinities as suggested by MD simulations (data not shown). It can be suggested that molecules of 1MI and 4MI bound at sites 1 and 2 mimic the inward and outward conformations of His64 (in wild-type CA II) and thus enhance the activity of the enzyme by aiding in proton shuttling. Similarly, molecules of I, 1MI, 2MI and 4MI bound to the active-site Zn^2+^ could well be responsible for inhibition. Inhibition of CA by 4MI binding close to the active-site metal ion has been reported before using NMR in which the catalytic zinc was replaced by catalytic cobalt (Elder *et al.*, 2005 ▶). However, site 3 in this study does not completely overlap with the site found by NMR studies.

These crystal structures show some ‘common binding sites’ in which at least two small imidazoles bind. It is these common sites that strongly hint that the binding is not random. It is interesting to note that two bound molecules of I share common binding sites (sites 6 and 7) with 4MI even though these sites do not appear to offer substantial binding interactions (Tables 2 ▶ *a* and 2 ▶ *d*). Similarly, bound on the surface near Ile91 at site 5, both 1MI and 4MI exhibit a dual conformation. Although this site is outside on the surface and it is unlikely that it can affect the catalytic efficiency of the enzyme, the presence of both compounds in almost the same orientation with a dual conformer is interesting (Tables 2 ▶ *b* and 2 ▶ *d*). Most binding sites (on and around the surface of the protein) could be considered unproductive and inconclusive, but the three sites (sites 1–3) within the active site strongly indicate activity enhancement and inhibition. In contrast, a different perspective could also be drawn. The data show that there are so many binding sites for imidazoles that every one of them makes a small contribution, which is why Duda and coworkers did not observe significant effects on activity enhancement when a single binding site (site 2 in this study) was destroyed by mutagenesis (Duda *et al.*, 2001 ▶). It should be noted that the kinetic data and crystallographic data do not fully correlate with each other in terms of the order in which these imidazoles enhance the activity of H64A CA II. The reason behind this mismatch could be attributed to the fact that all of the crystals were obtained at pH 8.0 while the kinetics studies were performed at the respective p*K*_a_ values of the imidazoles.

In addition to the inhibition of CA by these imidazoles, the presence of such a large number of binding sites also provides insights and preliminary data required for the fragment-addition approach of drug design, as we have previously suggested (Aggarwal, Kondeti *et al.*, 2013*b* ▶). For decades, CA has been a drug target for the treatment of various diseases, and a myriad of inhibitors are currently used as clinical drugs (Aggarwal & McKenna, 2012 ▶; Aggarwal, Kondeti *et al.*, 2013*a* ▶). However, most of these are sulfa drugs with a sulfon­amide functional group, to which almost 5% of the human population are allergic. As seen in this study, sulfur-lacking imidazoles can inhibit CA as well and help to circumvent the sulfa-allergy problem. Another unresolved issue in the field of CA inhibition is the unavailability and/or smattering of isoform-specific CA inhibitors (CAIs; Aggarwal, Kondeti *et al.*, 2013*b* ▶). Most CAIs known today inhibit more than one isoform of CA, which causes undesired side effects. Although the binding sites relevant for drug design (sites 1–4) reported here are very similar among the human catalytic CAs, the fragment-addition approach could be combined with a tail approach to design more isoform-specific inhibitors, as discussed previously (Aggarwal, Kondeti *et al.*, 2013*b* ▶). This approach offers a great opportunity, as is shown by carbohydrate (glycerol; Aggarwal, Boone *et al.*, 2013 ▶) binding near the active site of CA isoforms.

## Supplementary Material

PDB reference: H64A CA II, complex with imidazole, 4hf3

PDB reference: complex with 1-methylimidazole, 4hez

PDB reference: complex with 2-methylimidazole, 4hew

PDB reference: complex with 4-methylimidazole, 4hey

Supporting information. DOI: 10.1107/S2052252514004096/dc5001sup1.pdf

## Figures and Tables

**Table 1:** X-ray crystallographic data-collection and refinement statistics

	I	1MI	2MI	4MI
PDB code	4hf3	4hez	4hew	4hey
Data-collection statistics†				
Temperature (K)	100			
Wavelength (Å)	0.9	0.9	1.54	0.9
Space group	*P*2_1_			
Unit-cell parameters				
*a* (Å)	42.4	42.3	42.3	42.4
*b* (Å)	41.5	41.6	41.2	41.3
*c* (Å)	71.9	71.9	71.9	71.9
β (°)	104.4	104.5	104.4	104.4
Reflections				
Theoretical	86425	53643	26628	43032
Measured	85561	52034	26336	40407
Resolution (Å)	20.0–1.1 (1.19–1.15)	20.0–1.3 (1.40–1.35)	20.0–1.7 (1.76–1.70)	20.0–1.4 (1.50–1.45)
*R*_merge_ ‡ (%)	8.6 (46.8)	6.3 (37.9)	6.3 (46.2)	7.5 (39.4)
〈*I*/σ(*I*)〉	13.9 (2.8)	16.0 (3.3)	22.8 (4.2)	13.7 (3.1)
Completeness (%)	99.0 (97.9)	97.0 (96.2)	98.9 (97.2)	93.9 (95.1)
Multiplicity	3.6 (3.5)	3.5 (3.5)	6.6 (6.4)	3.3 (3.2)
Final model statistics				
*R*_cryst_ § (%)	15.8	15.1	16.5	15.2
*R*_free_ ¶ (%)	17.2	17.0	20.5	18.5
Residue Nos.	4–261			
No. of atoms††				
Protein	2385	2317	2311	2323
Ligand‡‡	45 (9)	48 (7)	12 (2)	54 (8)
Water	307	264	146	218
R.m.s.d.				
Bond lengths (Å)	0.01	0.01	0.01	0.01
Angles (°)	1.40	1.42	1.54	1.45
Ramachandran statistics (%)				
Favored	88.4	87.0	87.5	90.3
Allowed	11.6	13.0	12.5	9.8
Outliers	0.0	0.0	0.0	0.0
Average *B* factors (Å^2^)				
Main chain	9.11,	12.09	17.08	9.36
Side chain	11.68	15.14	19.98	11.99
Ligand	24.50	25.98	19.19	20.81
Solvent	21.90	24.90	26.07	21.93

† Values in parentheses are for the highest resolution bin.

‡ *R*_merge_ = 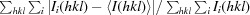 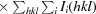.

§ *R*_cryst_ = 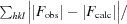 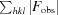 × 100.

¶ *R*_free_ is calculated in same manner as *R*_cryst_, except that it uses 5% of the reflection data that were omitted from refinement.

†† Includes alternate conformations.

‡‡ Values in parentheses represent the number of ligands bound in the structure.

**(a):** Imidazole (I).

	Chain ID, *B* factor (Å^2^), occupancy	BSA (Å^2^)	Hydrogen bonds, Δ*G* (kcal mol^−1^)
Binding sites inside the structure			
Site 3 (directly bound to Zn^2+^)	*C*, 12.4, 0.96	160.3	0, −12.4
Site 6 (Tyr7, Gly12, Pro13, Asp243, Trp245, Pro247)	*F*, 24.6, 0.92	149.1	2, −0.4
Binding sites on the surface			
Site 7 (Thr55, Leu57, Asn71)	*G*, 26.3, 0.98	78.7	0, −0.6
Site 9 (Asn130, Phe131, Gly132)	*N*, 27.5, 0.87	84.0	1, −0.6
Site 10 (Asn180, Arg182, Gly183)	*H*, 23.2, 0.98	69.5	0, −0.5
Site 11 (Thr37, Lys39, Gln255, Ile256)	*M*, 24.1, 0.87	129.6	1, 0.0
Site 12 (Phe131, Val135, Leu198, Pro202)	*I*, 27.4, 1.00	104.0	0, −1.1
Site 13 (Leu60, Asn61, Asn62, Gly171)	*K*, 31.0, 0.60	107.9	2, −0.3
Site 16 (Val49, Arg182, Leu185)	*E*, 24.0, 0.93	77.7	1, −0.1

**(b):** 1-Methylimidazole (1MI).

	Chain ID, *B* factor (Å^2^), occupancy	BSA (Å^2^)	Hydrogen bonds, Δ*G* (kcal mol^−1^)
Binding sites inside the structure			
Site 1 (His64 ‘in’ conformation)	*F*, 26.6, 0.92	124.4	0, −0.1
Site 2 (His64 ‘out’ conformation)	*C*, 26.7, 1.00	169.5	0, −0.5
Site 4 (Gly92, His94, Val121, Phe131, Leu198, Thr199, Thr200, Pro201, Pro202)	*E*, 12.2, 0.81	198.2	1, −0.7
Site 5 (Gln69, Phe70, Asp72, Ile91)	*G*, 25.4, dual conformation	110.7	0, −0.4
Binding sites on the surface			
Site 9 (Ile91, Asp130, Phe131, Gly132)	*J*, 31.9, 0.88	106.6	0, −0.8
Site 14 (Tyr51, Asp52, Ala54, Asn178, Phe179, Asp180, Pro181, Arg182)	*H*, 27.8, 0.92	152.8	0, 0.1
Site 15 (Lys112)	*L*, 32.0, 0.94	84.9	0, −0.4

**(c):** 2-Methylimidazole (2MI).

	Chain ID, *B* factor (Å^2^), occupancy	BSA (Å^2^)	Hydrogen bonds, Δ*G* (kcal mol^−1^)
Binding sites inside the structure			
Site 3 (directly bound to Zn^2+^)	*B*, 16.3, 1.00	25.5	4, −14.9
Site 4 (Gly92, His94, Val121, Phe131, Leu198, Thr199, Thr200, Pro201, Pro202)	*C*, 22.1, 1.00	164.8	1, −0.5

**(d):** 4-Methylimidazole (4MI).

	Chain ID, *B* factor (Å^2^), occupancy	BSA (Å^2^)	Hydrogen bonds, Δ*G* (kcal mol^−1^)
Binding sites inside the structure			
Site 1 (His64 ‘in’ conformation)	*F*, 20.6, 0.87	135.7	0, −0.1
Site 2 (His64 ‘out’ conformation)	*H*, 18.1, 0.95	171.0	1, 0.2
Site 3 (directly bound to Zn^2+^)	*E*, 9.7, 0.95	152.6	3, −13.5
Site 4 (Gly92, His94, Val121, Phe131, Leu198, Thr199, Thr200, Pro201, Pro202)	*G*, 29.0, 0.93	147.6	1, −0.6
Site 5 (Gln69, Phe70, Asp72, Ile91)	*P*, 22.9, dual conformation	69.1	0, −0.2
Site 6 (Tyr7, Gly12, Pro13, Asp243, Trp245, Pro247)	*C*, 24.8, 0.87	165.4	1, −0.4
Binding sites on the surface			
Site 7 (Thr55, Leu57, Asn71)	*K*, 25.9, 0.91	89.3	0, −0.6
Site 8 (Pro195, Thr208)	*J*, 11.3, 0.75	140.0	1, −0.5
