# Supplementary material for: Structural insight into activity enhancement and inhibition of H64A carbonic anhydrase II by imidazoles
Source: IUCrJ. 2014 Feb 28;1(Pt 2):129–35. doi: 10.1107/S2052252514004096 (PMC4062085; doi:10.1107/S2052252514004096)

IUCrJ

Volume 1 (2014)

Supporting information for article:

Structural insight into activity enhancement and inhibition of H64A carbonic anhydrase II by imidazoles

Mayank Aggarwal, Bhargav Kondeti, Chingkuang Tu, C. Mark Maupin, David N. Silverman and Robert McKenna

Supporting Information

Structural Insights into Activity Enhancement and Inhibition of H64A Carbonic Anhydrase II by Imidazoles

[1]Mayank Aggarwal, [1]Bhargav Kondeti, [3]Chingkuang Tu, [2]C. Mark Maupin, [3]David N. Silverman, and [1]Robert McKenna[*]

[1]Department of Biochemistry and Molecular Biology, PO Box 100245, University of Florida, Gainesville, FL 32610

[2]Department of Chemical and Biological Engineering, 1500 Illinois St, Colorado School of Mines, Golden, CO 80401

[3]Department of Pharmacology and Therapeutics, PO Box 100247, University of Florida, Gainesville, FL 32610

[*]Corresponding author:

Email: rmckenna@ufl.edu

Phone (office): 352-392-5696

Keywords: human carbonic anhydrase, H64A, activity enhancement, rescue, activation, imidazole.

S1. Methods

S1.1. Enzyme kinetics measurements

^{18}O exchange assay was carried out to study the kinetics of the catalyzed reaction of the hydration and dehydration of CO_2 and HCO_3^- -and how these small imidazoles (I, 1MI, 2MI and 4MI) affect the activity of H64A CA II. The pH values were chosen based on the pK_a of these imidazoles.

The method (Tu et al., 1989) relies on the depletion of ^{18}O from species of CO_2 as measured by membrane inlet mass spectrometry using an Extrel EXM-200 mass spectrometer (Pastorek et al., 1994). In the first stage of catalysis, the dehydration of labeled bicarbonate has a probability of labeling the active site with ^{18}O (eq 1). In a following step, protonation of the zinc-bound ^{18}O-labeled hydroxide results in the release of $H_2{}^{18}O$ to the solvent and loss of signal from the isotopic species (eq 2).

$$HCOO^{18}O^- + EZnH_2O \rightleftarrows EZnHCOO^{18}O^- \rightleftarrows COO + EZn^{18}OH^- \quad (1)$$

$$H^+His64\text{-}EZn^{18}OH^- \rightleftarrows His64\text{-}EZnH_2{}^{18}O \rightarrow His64\text{-}EZnH_2O + H_2{}^{18}O \quad (2)$$

This approach yields two rates: The R_1, the rate of CO_2 and HCO_3^- interconversion at chemical equilibrium (eq 1), as shown in Equation 3, and R_{H2O}, the rate of release from the enzyme of water with labeled substrate oxygen (eq 2).

$$R_1/[E] = k_{cat}^{ex} [CO_2]/(K_{eff}^{CO2} + [CO_2]) \quad (3)$$

In Equation 3, k_{cat}^{ex} is a rate constant for maximal interconversion of CO_2 and bicarbonate, K_{eff}^{CO2} represents a binding constant for the substrate to enzyme. The ratio $k_{cat}^{ex}/K_{eff}^{CO2}$ is considered equivalent in value to k_{cat}/K_M from steady state experiments, and is a measure of the successful binding and interconversion of substrate and product.

The second rate, R_{H2O}, is the component of the ^{18}O exchange that is dependent upon the donation of protons to the ^{18}O-labeled zinc-bound hydroxide. In such a step, His64 as a predominant proton donor in the catalysis provides a proton (Equation 2). The value of R_{H2O} can be determined and considered as the rate constant for proton transfer from His64 to the zinc-bound hydroxide according eq 4, in which k_B

is the rate constant for proton transfer to the zinc-bound hydroxide and $(K_a)_{donor}$ and $(K_a)_{ZnH2O}$ are ionization constants of the proton donor, His64, and zinc-bound water. The least-squares determination of kinetic constants of Equation 3 and Equation 4 was carried out using Enzfitter (Biosoft).

$$R_{H2O}/[E] = k_B/([1 + (K_a)_{donor} /[H^+]][1 + [H^+]/(K_a)_{ZnH2O}]) \qquad (4)$$

The uncatalyzed and carbonic-anhydrase-catalyzed exchanges of ^{18}O between CO_2 and water at chemical equilibrium were measured in the absence of buffer (to prevent interference from the second intermolecular proton transfer reaction) at a total substrate concentration of 25 mM and 25 °C.

References

Pastorek, J., Pastoreková, S., Callebaut, I., Mornon, J. P., Zelník, V., Opavský, R., Zat’ovicová, M., Liao, S., Portetelle, D., & Stanbridge, E. J. (1994). *Oncogene*. **9**, 2877–2888.

Tu, C., Silverman, D. N., Forsman, C., Jonsson, B. H., & Lindskog, S. (1989). *Biochemistry (Mosc.)*. **28**, 7913–7918.

Figure S1 (a) The effect of imidazole (I) on R1/[E] (black) and R_{H2O}/[E] (red) catalyzed by H64A CA II. (b) Crystal structure of H64A CA II in complex with imidazole, showing the site of inhibition (site 3). His64 from wt-CA II is superposed and shown as yellow sticks for orientation perspective.

(a)

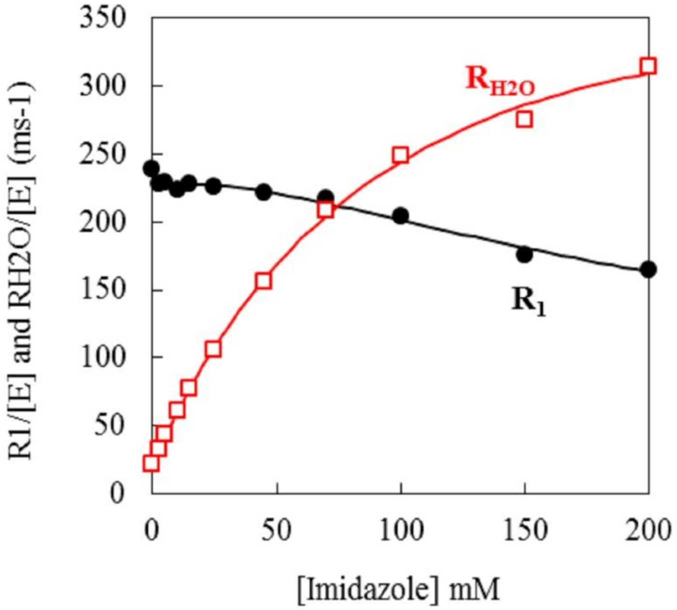

(b)

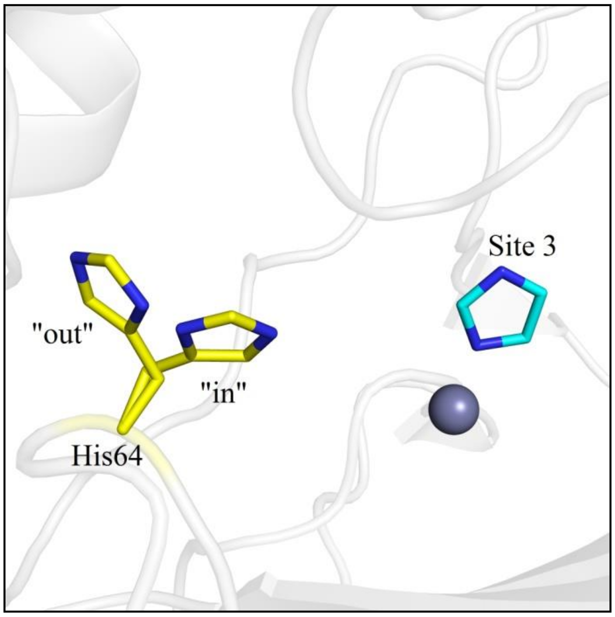

Figure S2 (a) The effect of 2-methyl imidazole (2MI) on R1/[E] (black) and R_{H2O}/[E] (red) catalyzed by H64A CA II. (b) Crystal structure of H64A CA II in complex with 2-methyl imidazole, showing the sites of inhibition (site 3 and most likely also site 4). His64 from wt-CA II is superposed and shown as yellow sticks for orientation perspective.

(a)

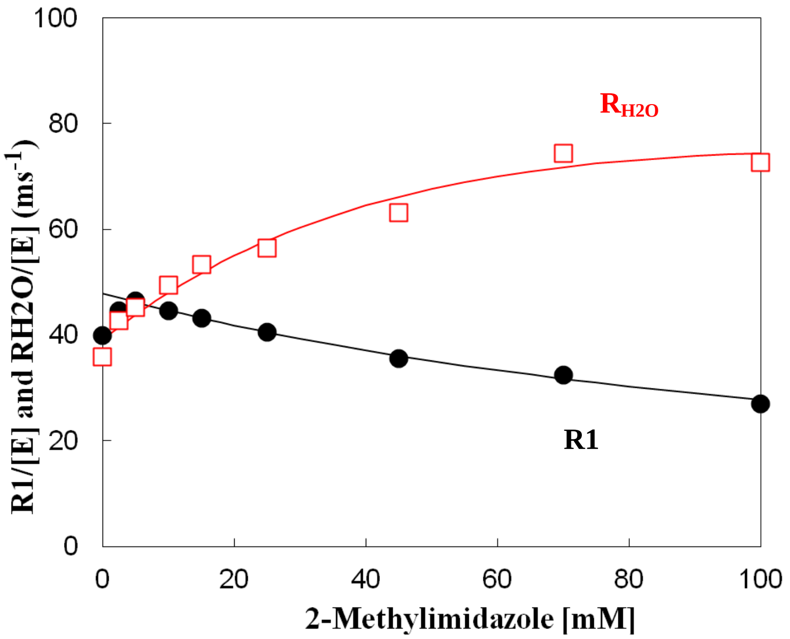

(b)

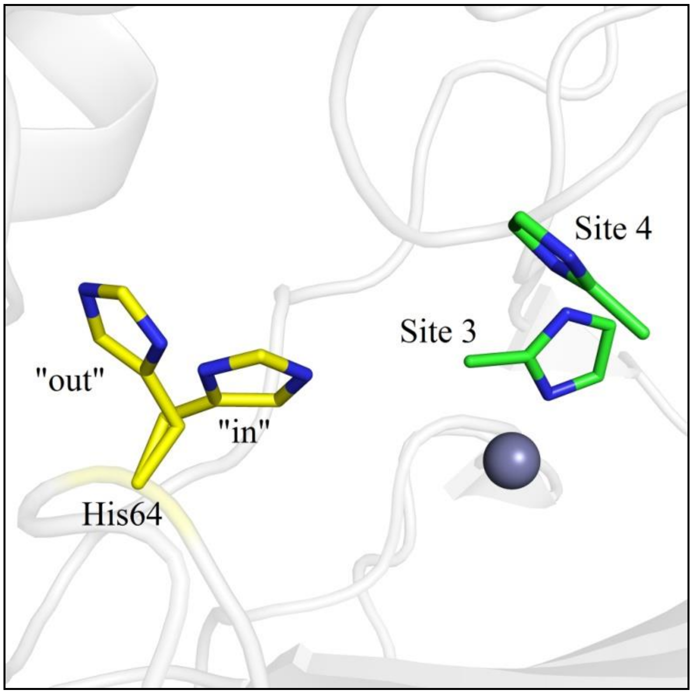

Figure S3 The activity enhancement by exogenous proton donors of R_{H2O}/[E] (s^{-1}) catalyzed by H64A CA II. The proton donors were I (black) at pH 6.7, 1MI (red) at pH 7.3, 2MI (blue) at pH 8.1, and 4MI (green) at pH 7.7. The data were obtained at 25 °C using solutions maintained at a minimal ionic strength of 0.2 M by addition of Na_2SO_4.

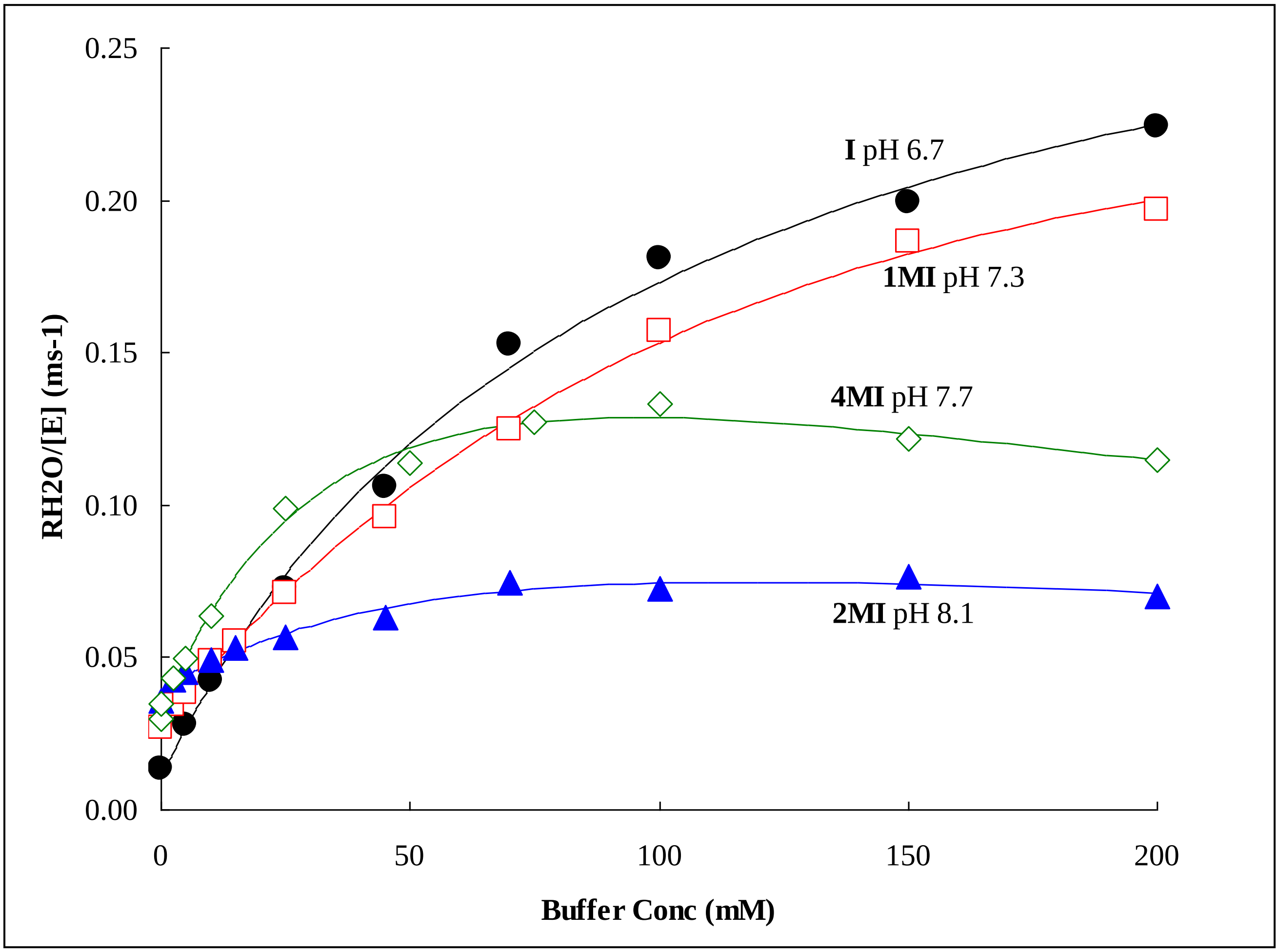

Supplement: Supplementary file 1 [file m-01-00129-sup1.pdf]
